# Silencing of *LOC389641* impairs cell proliferation and induces autophagy via EGFR/MET signaling in lung adenocarcinoma

**DOI:** 10.18632/aging.202286

**Published:** 2020-12-09

**Authors:** Lei Xiao, Yu Li, Xiaofei Zeng, Zhiqing Zhou, Shengmin Hu, Shenglin Zhang, Yi Zhou, Zhan Zhang, Han Zhao, Huijie Zhao, David G. Beer, Rui Mao, Guoan Chen

**Affiliations:** 1 Cancer Center of the First Affiliated Hospital, Xinjiang Medical University, Urumqi, China; 2 School of Medicine, Southern University of Science and Technology, Shenzhen, China; 3 Department of Surgery, University of Michigan Medical School, Ann Arbor, MI 48109, USA

**Keywords:** non-small cell lung cancer (NSCLC), long non-coding RNA (lncRNA), LOC389641, prognosis, cell proliferation

## Abstract

High-throughput RNA-sequencing studies of tumor samples have identified a large number of long non-coding RNAs (lncRNAs) which are associated with various types of cancer. LncRNAs play key roles in regulating chromatin dynamics, gene expression, growth, differentiation and development. However, the role of *LOC389641* in non-small cell lung cancer (NSCLC) tumorigenesis is not clear. Here, we investigated the expression pattern, roles and mechanism of *LOC389641* in lung cancer. *LOC389641* expressions in tumor tissues and cell lines were measured by qRT-PCR. Functional studies including colony formation, cell proliferation and invasion were performed in lung cancer cell lines and Western blot was used to exam the protein changes upon siRNA treatment. We found that *LOC389641* was highly expressed in lung adenocarcinomas and was associated with poor patient survival. Silencing of *LOC389641* reduced colony formation, cell proliferation and invasion, as well as induced autophagy and apoptosis of lung adenocarcinoma cell lines *in vitro*. Mechanistically, downregulation of *LOC389641* was found to decrease EGFR, MET and STAT3 proteins expression in lung cancer cells. *LOC389641* is highly expressed and plays an oncogenic role in this type of NSCLC. Because of its specificity, *LOC389641* may be a potential biomarker for prognosis and a possible target for lung adenocarcinoma.

## INTRODUCTION

Lung cancer is the most frequently diagnosed cancer and the leading cause of cancer-associated death worldwide [1, 2]. Non-small cell lung cancer (NSCLC), which is the predominant form and accounts for 80–85% of all lung cancers [3], includes lung squamous cell carcinoma (SCC), lung adenocarcinoma (AD) and large-cell carcinoma (LCC). Although much progress in various therapies has been made for NSCLC patients in the past decade, such as EGFR and PD-L1 targeted therapy, the prognosis of NSCLC patients remains poor [1, 2]. Thus, it is critical to understand the underlying pathological mechanisms and identify novel diagnostic/prognostic markers for NSCLC in order to develop new therapeutic targets to increase patient survival.

Previous studies have suggested that long non-coding RNAs (lncRNAs) not only play an important role in regulating gene expression at various levels, but also can function as oncogenes or tumor suppressor genes, and thus are directly related with the malignant biological behavior of multiple tumors [4, 5]. Accumulating evidence indicate that lncRNAs can serve as novel molecular biomarkers for specific processes in cancer biology, including cell growth, death, cell cycle progression, proliferation, invasion and migration [5].

Though studies have shown that lncRNAs play important roles in gene expression and cancer progression, their role in NSCLC remains largely unexamined. To investigate the expression pattern and importance of lncRNAs in NSCLC, we analyzed three large sets of RNAseq data including TCGA [6], Seo [7] and UM [8], and discovered *LOC389641* as one of the most up-regulated lncRNAs as compared to normal lung tissues. A previous study indicated that *LOC389641* can increase cell invasion of pancreatic ductal adenocarcinoma cells by regulating E-Cadherin expression [9]. Our current study was aimed at examining the relationship between *LOC389641* expression and its clinical significance in NSCLC, and defining its potential oncogenic functions such as effects upon NSCLC cell proliferation, cell death, migration and invasion.

## RESULTS

### *LOC389641* is highly expressed in lung adenocarcinomas and associated with poor patient survival

**Figure 1:**
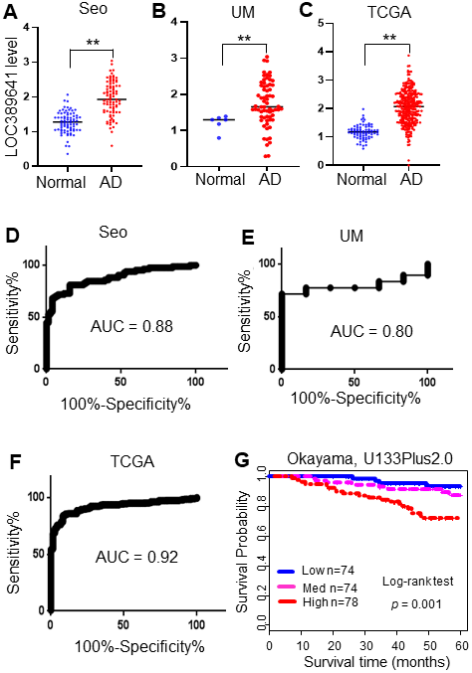
***LOC389641* is highly expressed in lung adenocarcinomas and is associated with poorer patient survival.** (**A**–**C**), Dot plots of *LOC389641* expression levels in lung adenocarcinoma (AD) and normal lung (N) tissues in the Seo (85 AD vs 77 N), TCGA (312 AD vs 79 N) and UM (67 AD vs 6 N) RNAseq datasets (y-axis is log2 of FPKM value, ** AD vs. Normal, t test, *p <* 0.01). (**D**–**F**) ROC curves with AUC values of *LOC389641* in Seo (85 AD vs 77 N), TCGA (312 AD vs 79 N) and UM (67 AD vs 6 N) RNAseq datasets. (**G**) Kaplan-Meier survival curve with log-rank test of *LOC389641* in Okayama dataset (226 ADs, U133plus2.0 array). Higher *LOC389641* expression (1/3 cases for each group based on *LOC389641* value) was significantly correlated with poor patient survival.

In our previous study, we combined the gene expression data from three large RNAseq datasets and identified differentially expressed lncRNAs in lung adenocarcinomas (AD) [10]. The three cohorts were the Korean cohort (Seo) [7], including 85 ADs and 77 normal lung tissues; the Cancer Genome Atlas (TCGA) data [6], including 312 ADs and 79 normal lung tissue samples; and the University of Michigan (UM) cohort, including 67 ADs and 6 normal lung tissue samples [8]. Among the lncRNAs dysregulated in ADs, we found that *LOC389641* was highly expressed in ADs compared with normal lung tissues in all three cohorts (Figure 1A–1C). In order to determine whether the *LOC389641* expression pattern has significant clinical utility, we performed a Receiver Operating Characteristic (ROC) curve analysis. The Area Under Curve (AUC) values were larger than 0.80 in all 3 cohorts (Figure 1D–1F) indicating that *LOC389641* may be potentially used as a novel diagnostic marker for this type of lung cancer. We next analyzed the association of *LOC389641* and patient survival using the Okayama microarray dataset [11] containing 224 early stage (stage 1 and 2) AD tissue samples. Kaplan-Meier analysis revealed that the patients with elevated *LOC389641* expression demonstrated a poorer overall survival as compared to those patients whose tumors had low expression (p = 0.001) (Figure 1G).

### Validation of *LOC389641* expression in an independent cohort of ADs and lung cancer cell lines

**Figure 2:**
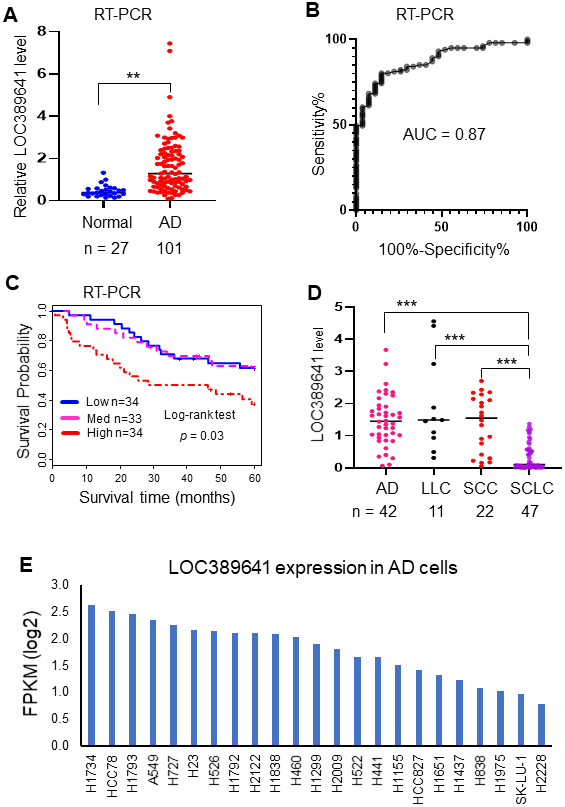
**Validation of *LOC389641* expression in an independent cohort of ADs and lung cancer cell lines.** (**A**) Dot plot indicating *LOC389641* expression was higher in ADs in an independent validation cohort measured by RT-PCR. y-axis is fold-change to mean of all tissues, ** AD vs. normal, t test, *p* < 0.01. (**B**) ROC curve indicated an excellent AUC (0.87) for classifying 101 AD from 27 normal lung tissues based on *LOC389641* expression in this independent validation cohort. (**C**) Kaplan-Meier survival curve indicated higher *LOC389641* expression was unfavorable for patient survival in this independent validation cohort. Log-rank test, *p* = 0.03. (**D**) *LOC389641* expression in different types of lung cancer cell lines. *LOC389641* expression was significantly lower in small cell lung cancer (SCLC) cell lines (CCLE, RNAseq data, *** NSCLC vs. SCLC, *p* < 0.001 by t test). (**E**) *LOC389641* expression in individual lung adenocarcinoma cell lines (CCLE, RNAseq data) and these cell lines were ranked in order of *LOC389641* expression.

In order to validate the association between *LOC389641* expression and survival discovered from the above analyzed RNAseq and microarray datasets, we performed qRT-PCR in an independent cohort of tissue samples from UM including 101 AD and 27 normal lung tissues. We found that *LOC389641* was significantly increased in ADs (vs normal lung tissues) (*p* < 0.01, Figure 2A). ROC curve analysis revealed the AUC value was 0.87 thus indicating *LOC389641* expression could significantly separate the ADs from normal lung tissues (Figure 2B). Kaplan-Meier survival curve analysis indicated that higher *LOC389641* expression was unfavorable for patient survival in this validation cohort (Figure 2C).

Next, we want to determine whether *LOC389641* expression was associated with specifics type of lung cancer cell lines which are available through analysis of the CCLE database [12]. We found *LOC389641* expression was significantly lower in small cell lung cancer (SCLC) cell lines (Figure 2D). We also found *LOC389641* was expressed in a variety of AD cell lines (Figure 2E). Taken together, this data indicated that *LOC389641* was highly expressed in AD and other types of NSCLC. These results support the potential of *LOC389641* as a biomarker for NSCLC.

### LOC389641 cellular location and knockdown impairs cell proliferation

**Figure 3:**
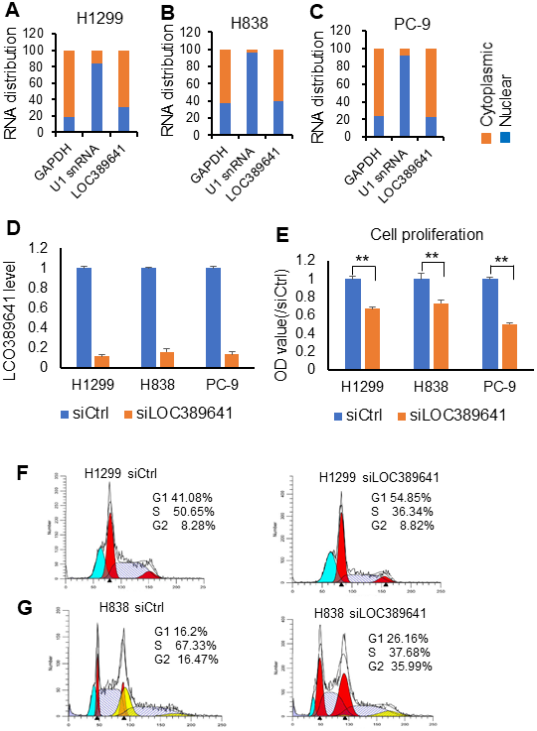
***LOC389641* cellular location and knockdown impairs cell proliferation.** (**A**–**C**) qRT-PCR showing the nuclear and cytoplasmic fractions of *LOC389641* in H1299, H838 and PC-9 cells. GAPDH is used as cytoplasmic control and U1 snRNA as nuclear control. *LOC389641* is primarily in cytoplasm (61% - 77%). (**D**) *LOC389641* siRNA knockdown efficiency (48 h) in H1299, H838 and PC-9 cell lines measured by qRT-PCR. GAPDH is used as loading control. (**E**) Cell proliferation is decreased after *LOC389641* siRNAs treatment in H1299, H838 and PC-9 cell lines. ** *p* < 0.01 by t test. (**F**, **G**) Flow cytometry analysis to evaluate the effects of *LOC389641* on cell cycle distribution in H1299 and H838 cell lines.

To examine the potential cellular location of *LOC389641*, we assessed its expression in both nuclear and cytoplasmic fractions from H1299, H838 and PC-9 cells using qRT-PCR, and found that 61%-77% of the expression of *LOC389641* was primarily localized in cytoplasm (Figure 3A–3C).

In order to reduce off-target effects by siRNAs, we used SMARTpool siRNAs of *LOC389641* which represent a mixture of 4 individual siRNAs. *LOC389641* expression decreased significantly following siRNAs transfection indicating that the siRNAs had the high interference efficiency in these cells (Figure 3D). We next evaluated the effects of *LOC389641* on cell proliferation using WST-1 assay in AD cells. Our data revealed that knockdown of *LOC389641* significantly suppressed the cell proliferation of three AD cell lines (Figure 3E). Cell cycle analysis of flow cytometry showed that more cells were arrested at G1 phrase with less in S phrase in the siRNAs (siLOC389641) group as compared to the control (siCtrl) group (Figure 3F, 3G). Taken together, these results suggest that *LOC389641* is cytoplasmic and its knockdown can influence the cell cycle and cell proliferation.

### *LOC389641* knockdown impairs colony formation and invasion of lung cancer cells

**Figure 4:**
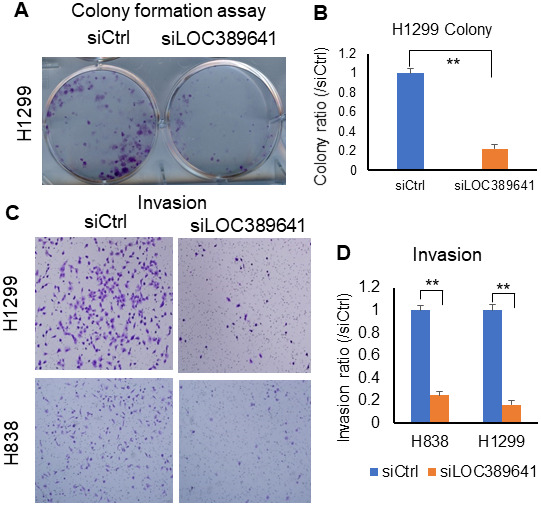
***LOC389641* knockdown impairs colony formation and invasion in lung cancer cells.** (**A**, **B**), Colony formation is decreased after *LOC389641* knockdown with siRNAs on H1299 cell line. (**B**) is the relative quantified value. ** *p* < 0.01 by t test. (**C**, **D**), Silencing of *LOC389641 by* siRNAs decreased cellular invasion ability in H1299 and H838 cell lines (10X). (**D**) are the relative quantified value. ** *p* < 0.01 by t test.

As shown in Figure 4A, 4B, cell colony formation was substantially decreased in *LOC389641* siRNA-treated cells (H1299) (siLOC389641) (*p* < 0.01), suggesting that knockdown of *LOC389641* influences colony-forming ability in ADs. Therefore, we believed that knockdown of *LOC389641* could be the important target to reduce the cell growth ability of ADs. The transwell invasion assay is a commonly used for the effective analysis of a cell’s invasion ability. Transwell invasion assays showed that cell invasion was inhibited after *LOC389641* siRNA transfection as compared to control group (siCtrl) for both H1299 and H838 cells (Figure 4C, 4D). (*p* < 0.01). These results suggest that *LOC389641* gene silencing decreases the number of cells invading the membrane, and also implying *LOC389641* may promote lung cancer spread and metastasis to distance organs.

### Knockdown of *LOC389641* causes apoptosis and autophagy in lung cancer cell lines

**Figure 5:**
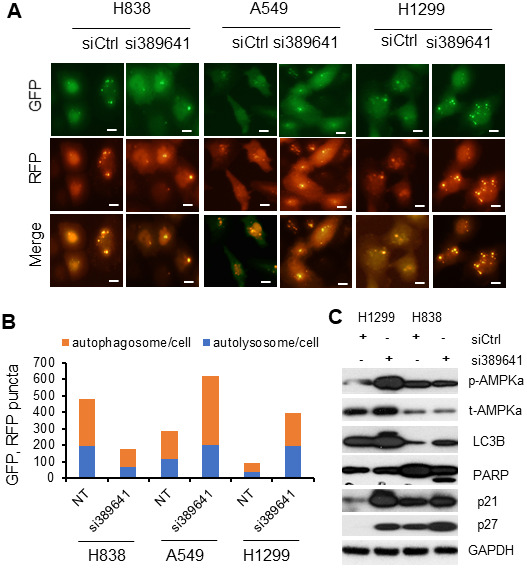
***LOC389641* knockdown induces autophagy and apoptosis in lung cancer cell lines.** (**A**, **B**) H1299, A549 and H838 were treated with *LOC389641* siRNAs for 48h and transfected with Premo Autophagy Tandem Sensor RFP-GFP-LC3B for 24h. Cells were visualized live using a fluorescence microscope. Autophagosomes and autolysosomes in each 200X field were counted, at least 100 cells were counted for each siRNA treatment per cell line. Autophagic flow was increased upon *LOC389641* silencing in A549 and H1299 cells but were decreased in H838 cells. Scale bar: 5 μm. (**C**) Western blot showing the changes of autophagy, apoptosis and cell cycle related proteins upon *LOC389641* silencing in H1299 and H838 cells (*LOC389641* siRNAs treated for 72 h). Apoptosis marker (cleaved-PARP) was induced upon *LOC389641* knockdown in H838 cell line.

To investigate whether *LOC389641* affects cell death signaling, we examined cell autophagy and apoptosis following *LOC389641* knockdown. For monitoring autophagy flux after *LOC389641* siRNAs transfection, we introduced the chimeric construct RFP-GFP-LC3B into H838, H1299 and A549 cells. We observed that the number of RFP-LC3B puncta (autolysosomes) and merged puncta (autophagosomes) were increased in *LOC389641* siRNA-transfected H1299 and A549 cells, whereas they were decreased in H838 cells (Figure 5A, 5B). These results suggest that knockdown of *LOC389641* can induce autophagy in A549 and H1299 cells, while inducing autophagy in H838 cells. To more specifically define the effects of silencing of *LOC389641* and the role in lung cancer autophagy, we investigated the autophagy-related proteins, AMPK and LC3B, by Western blotting. We found that phosphorylated AMPK was significantly increased in H1299 after *LOC389641*-siRNA knockdown, but not in H838. AMPK is a critical regulator of autophagy and the activation of AMPK results in enhanced autophagy [13]. Further, LC3 lipidation, which is indicated by the formation of LC3-II, is an index of autophagy. *LOC389641* siRNA significantly increased LC3 lipidation in H1299 cells but not in H838 cells (Figure 5C). This result may explain the differences in autophagy signaling after *LOC389641* siRNA knockdown in H1299 (autophagy-induced cell line) vs H838 (non-autophagy-induced cell line).

Next, we measured PARP, a marker of apoptosis, by Western blot. We found that cleaved-PARP was decreased with a cleaved band of lower molecular weight occurring following siRNA interference in H838 cells. In the majority of cases, apoptosis and autophagy are mutually inhibitory [14]. For cell death pathways upon *LOC389641* knockdown, H838 undergoes apoptosis, while in H1299 it induces autophagy signaling. Finally, we found the expression of cell cycle inhibitory proteins p21 and p27 protein were increased after *LOC389641* knockdown. This is consistent with the data shown in Figure 3F, 3G, where the inhibition of *LOC389641* caused G1 phase arrest in H1299 and H838 cells.

### *LOC389641* knockdown decreases EGFR, MET and STAT3 proteins expression

**Figure 6:**
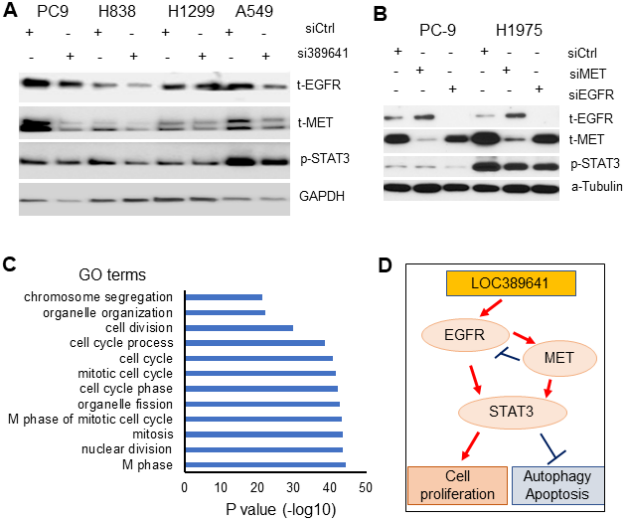
***LOC389641* knockdown decreases EGFR, MET and STAT3 proteins expression.** (**A**) Western blot showing total EGFR, total MET and phosphor-STAT3 proteins were decreased upon *LOC389641* silencing (si389641) in H1299 and H838 cells. *LOC389641* siRNA treatment was for 72 h. GAPDH was uses as protein loading control. (**B**) Western blot showing the changes of EGFR, MET and STAT3 proteins after EGFR or MET siRNAs treatment in PC-9 and H1975 cells. siRNAs treated for 72 h, α-Tubulin used as protein loading control. (**C**) Gene Ontology (GO) analysis of *LOC389641* positively-correlated genes. Cell cycle related biology processes were on the top of the list. (**D**) The schematic model of the cell proliferation, autophagy, apoptosis signaling regulated by MET, EGFR and STAT3 triggered by *LOC389641* knockdown in lung cancer.

To further define the mechanism underlying the effects of *LOC389641* on AD, we explored the expression status of proteins in major pathways of AD. Western blot showed that total EGFR, total MET and phosphorylated STAT3 proteins were decreased in AD cell lines (PC9, A549, H1299 and H838). These three proteins are the well-known major proto-oncogenes/proteins in AD [15–17] (Figure 6A).

To determine the relationship between EGFR and MET, we examined EGFR, MET and STAT3 proteins changes upon siRNAs treatment of EGFR or MET in two EGFR mutant AD cells, PC-9 and H1975. Western blot showed that MET protein was decreased upon EGFR knockdown, while EGFR protein was increased after MET knockdown indicating MET may be a downstream protein of EGFR (Figure 6B). Increased EGFR protein may provide the cell with a feedback mechanism upon MET knockdown. STAT3 was decreased upon EGFR or MET knockdown indicating STAT3 may be regulated by both EGFR and MET in AD cells.

Finally, we performed DAVID gene ontology analysis to explore potential underlying biological processes associated with *LOC389641*. We first did the Pearson correlation analysis between *LOC389641* and the 20,000 coding genes based on the Okayama data set [11]. There were 348 positively-correlated genes (n = 226, r = 0.46, *p* < 0.001) (Supplementary Table 1). Analysis of biological processes with DAVID using these 348 genes, revealed that cell cycle regulation-related biological processes were on the top of the list, which further confirms that *LOC389641* is involved in cell proliferation and cell cycle regulation (Figure 6C and Supplementary Table 2).

In summary, our analyses suggest that *LOC389641*-mediated control of cell proliferation, autophagy, apoptosis in AD may occur via EGFR, MET and STAT3 signaling (Figure 6D). Further mechanistic studies of the role of *LOC389641* in these signaling pathways are warranted.

## DISCUSSION

The results of our studies indicate that *LOC389641* plays an oncogenic role in NSCLC biology. We found that *LOC389641* is highly expressed in AD, and that increased expression is associated with poor patient survival. Using a ROC curve analysis, *LOC389641* levels significantly separate ADs from normal lung tissues. *LOC389641* expression in NSCLC appears to be higher than in small cell lung cancer (SCLC). Therefore, *LOC389641* may have potential use as a biomarker for NSCLC.

We found that knockdown of *LOC389641* significantly reduced cell proliferation, colony formation and invasion capability of lung cancer cells. Accelerating cell cycle processes is a major attribute of the unlimited proliferation and rapid growth characteristic of cancer cells [18]. Cell cycle progression is regulated by cyclin kinase regulators p21 and p27, which are upregulated during cell cycle arrest [19]. In this study, we found that p21 and p27 proteins were increased after *LOC389641* siRNA knockdown. Consistent with this, flow cytometry analysis indicated that inhibition of *LOC389641* expression caused G1 phase arrest in H1299 and H838 lung AD cell lines. DAVID gene ontology analysis corroborated that cell cycle signaling is associated with the genes correlated with highly expressed *LOC389641*. These data imply that *LOC389641* promotes tumor cell proliferation and cell growth through modulation of the cell cycle. The exact mechanisms how *LOC389641* regulates cell cycle progression requires further in-depth studies.

Autophagy and apoptosis occur when cells are under stress [14]. Autophagy has been found to be closely related to apoptosis [16]. Regulation of the balance between apoptosis and autophagy is critical during cell death. In some conditions, autophagy has a protective effect, preventing cells from undergoing apoptosis by promoting cell survival [20]. In others, excessive autophagy can induce cell death. In this study, knocking down *LOC389641* in H838 cells stimulated PARP cleavage, which is the hallmark of apoptotic cell death, but no such change was seen in H1299. Moreover, our data indicates autophagy occurred in H1299, but not in H838. Therefore, the type of cell death may be differed among lung AD cells after *LOC389641* siRNAs treatment. Autophagy occurred in H1299 which may have an anti-apoptotic role, but was then followed by apoptosis. Whether we can clearly understand the crosstalk between autophagy and apoptosis upon *LOC389641* silencing is an intriguing issue, which certainly will require further investigation.

MET, EGFR and STAT3 function as oncogenes in many types of malignant tumors including NSCLC. Studies have revealed that the crucial importance of these three genes/proteins in tumor cell survival, proliferation, apoptosis, autophagy, angiogenesis and metastasis in NSCLC [15–17, 21]. EGFR and MET are receptors tyrosine kinases [22]. Signaling interactions between MET and EGFR pathways have been reported [23, 24]. Both EGFR and MET use an overlapping repertoire of signaling adaptors and downstream effector pathways, highlighting their ability to co-drive oncogenic signaling, as has been observed in NSCLC models [25]. EGF-induces activation of extracellular signal–regulated kinase 1 (Erk1) and Erk2 via Ras, of Akt via phospholipase C γ and phosphatidylinositol 3-kinase (PI3K), and of signal transducer and activator of transcription 3 (STAT3) and STAT5 via Janus kinase 2 (JAK2) [26]. When the ligand for MET, the human hepatocyte growth factor (HGF), binds to MET, it can activate downstream RAS/ERK/MAPK, PI3K-AKT, Wnt/β-catenin, and STAT signaling pathways [16]. STAT3 protein becomes activated primarily by tyrosine phosphorylation including MET and EGFR. STAT3 is constitutively activated and often required to maintain the transformed phenotype in a majority of malignant tumors including lung cancer, functioning as an oncogene [16]. We found that MET, EGFR and phosphorylated STAT3 proteins were decreased after *LOC389641* knockdown in human lung AD cell lines. It is probable that EGFR and MET may act in concert to activate STAT3 and promote the malignant phenotype, or perhaps function independently (Figure 6D). Further research is required to identify the mechanisms how silencing of *LOC389641* influences NSCLC progression via oncogenes MET, EGFR and STAT3.

In conclusion, the present study demonstrates *LOC389641* as a potential oncogene which acts through influencing MET, EGFR and STAT3, thereby regulating multiple cell survival/death signals including cell proliferation, colony formation, cell invasion, apoptosis and autophagy. *LOC389641* has potential as a new diagnostic/prognostic marker, as well as a therapeutic target for lung cancer.

## MATERIALS AND METHODS

### Patients and tissue samples and lung cancer cell lines

We collected NSCLC tissues and adjacent normal tissues from surgical resections of patients between 1991 and 2012 at the University of Michigan Health System. The study was approved by the University of Michigan Institutional Review Board and Ethics Committee, and each patient provided written informed consent. All tissues were confirmed by histopathological evaluation. No patients received radiotherapy or chemotherapy prior to surgery. Pertinent clinical data, including age, sex, histologic tumor type, stage and differentiation were collected from surgical and pathological records. All tissues were collected, quickly frozen in liquid nitrogen and then stored at –80° C before RNA extraction. The median follow-up of living patients was 8.6 years. These samples were also used in our previous studies [10, 27].

The NSCLC cell lines (H1299, H838, A549, PC9) were purchased from the American Type Culture Collection. All cells were cultured in RPMI 1640, 10% FBS, 1% antibiotic-antimycotic at 37° C with 5% CO2. All cell lines were genotyped for identity at the University of Michigan Sequencing Core and were tested for Mycoplasma contamination routinely.

### RNA isolation and quantitative real-time PCR (qRT-PCR)

Total RNA was isolated from tissue samples and cell lines using miRNeasy Mini kit (Qiagen) according to the manufacturers’ instructions. The extracted RNAs were converted into cDNA by High-Capacity cDNA Reverse Transcription Kit (Applied Biosystems). The expression levels of *LOC389641* in NSCLC tissues and cell lines were measured by qRT-PCR using the SYBR-Green method (Takara). The oligonucleotide primers for *LOC389641* included: forward 5’-CCTGCCTGCGAAGAACTCC, and reverse 5’-ACCACACAACAGAAGAGCAGAA. Fold-change in expression was calculated relative to house-keeping genes (GAPDH or ACTB) and were further normalized to the median value of normal samples.

### siRNA-mediated knockdown of *LOC389641*

To down-regulate *LOC389641* expression, a SMARTpool of *LOC389641* siRNAs (Dharmacon) was used in this study together with non-targeting siRNA as controls (scribe siRNA #1 Dharmacon). For this assay, cells were plated in 96-well or 6-well plates and siRNAs or non-targeting controls transfected into cultured cells 24h after plating. Transfection was also performed with Lipofectamine® RNAiMax Reagent (Invitrogen, USA) in OptiMEM medium. Knockdown efficiency was determined by qRT-PCR.

### Cell proliferation assay

Proliferation was measured using the cell proliferation reagent, Water Soluble Tetrazolium (WST)-1 (Roche). For this assay, cells were seeded in 96 well plates. For non-targeting control (siCtrl) group and *LOC389641* knockdown (siLOC389641) group, cells were plated in triplicate and the siRNAs were transfected into cultured cells 24 h after plating. The cells proliferation rates were measured at 72 h after siRNAs transfection.

### Cell invasion assay

For invasion assays, cells were treated with the indicated siRNAs. After 48 h transfection, the cells (H1299: 0.5×10^5^; H838: 1×10^5^) were collected and resuspended in serum-free medium. These cells were seeded onto basement membrane matrix Boyden chambers (8-μm pore size, BD) present in the insert of a 24-well culture plate. The lower compartment of Boyden chambers was filled with 600 μl medium containing 20% FBS as chemoattractant. After 48h incubation, matrigel and non-migrating cells were removed from the top chamber with cotton swabs. Invading cells on the bottom were stained with Diff-QuikTM Stain Set (SIEMENS). After air drying, the number of invaded cells per chamber was counted in five separate fields under a microscope and the average values calculated.

### Cell cycle analysis by flow cytometry

Cells were collected 48 hours after transfection in cell cycle assay. H1299 and H838 cells were washed three times with cold phosphate buffer saline (PBS, 0.1 M, pH 7.4), fixed with 70% ice-cold ethanol at -20° C for 12 hours, washed with PBS, re-suspended in 1ml of propidium iodide (PI) staining solution, and incubated for 30 min in the dark at room temperature. Analyses were performed on FACScan flow cytometer.

### Western blotting analysis

Proteins were separated by electrophoresis through a 10% SDS/polyacrylamide gel. After electrophoresis, all proteins were transferred to PVDF membranes. Blocking was performed with 5% bovine serum albumin for 1 h. The membranes carrying transferred proteins were incubated with primary monoclonal antibodies at 4° C on orbital shaking overnight. After incubation with HRP-conjugated secondary antibody for 1 h at room temperature, the PVDF membranes were followed by ECL and exposed to X-ray film or the ChemiDoc MP Imaging System (BIO-RAD).

### Published microarray and RNA sequencing data collection

Three published RNAseq data sets from lung cancer tissues including Seo et al., (85 AD and 77 normal lung tissues) [7], TCGA (309 AD and 73 normal lung tissues) [6] and UM (67 AD and 6 normal lung tissues) [8]. RNAseq data of lung cancer cell lines were obtained from the CCLE database [12]. Expression levels of transcripts were represented as reads per kilobase per million mapped reads (RPKM) [28]. One published Affymetrix microarray data [11] representing 226 primary early stage lung ADs was also utilized. The CEL files of microarray data were normalized using the Robust Multi-array Average (RMA) method [29].

### Statistical analyses for experimental data

Statistical analyses were analyzed using the R packages and GraphPad Prism 8. Kaplan-Meier survival curves were used to display survival curves, and survival differences were evaluated by the log-rank test using R packages. The Area Under Curve (AUC) was determined by the Receiver Operating Characteristic (ROC) curve using GraphPad Prism 8. It was used to show the tradeoff between specificity and sensitivity for the different possible cut-points of a diagnostic test. The dot plots in Figure 1 and were drew by GraphPad Prism 8. The other data such as *LOC389641* expression level, proliferation, colony formation and invasion were evaluated by unpaired Student’s t-test. P value < 0.05 was considered to be statistically different.

## Supplementary Material

Supplementary Table 1

Supplementary Table 2
